# Therapies for bruxism: a systematic review and network meta-analysis (protocol)

**DOI:** 10.1186/s13643-016-0397-z

**Published:** 2017-01-13

**Authors:** Mauro Elias Mesko, Brian Hutton, Jovito Adiel Skupien, Rafael Sarkis-Onofre, David Moher, Tatiana Pereira-Cenci

**Affiliations:** 1 Graduate Program in Dentistry, Federal University of Pelotas, Rua Gonçalves Chaves, 457, 5th floor, Pelotas, RS Brazil; 2 Clinical Epidemiology Program, Ottawa Hospital Research Institute, The Ottawa Hospital, General Campus, 501 Smyth Rd, Ottawa, ON K1H8L6 Canada; 3 School of Epidemiology, Public Health and Preventive Medicine, University of Ottawa, Ottawa, ON Canada; 4 School of Dentistry, Franciscan University Center, Rua Silva Jardim, 1175, 6th floor, Santa Maria, RS 97010-491 Brazil

**Keywords:** Bruxism therapy, Sleep bruxism, Evidence-based dentistry

## Abstract

**Background:**

Bruxism is a sleep disorder characterized by grinding and clenching of the teeth that may be related to irreversible tooth injuries. It is a prevalent condition occurring in up to 31% of adults. However, there is no definitive answer as to which of the many currently available treatments (including drug therapy, intramuscular injections, physiotherapy, biofeedback, kinesiotherapy, use of intraoral devices, or psychological therapy) is the best for the clinical management of the different manifestations of bruxism. The aim of this systematic review and network meta-analysis is to answer the following question: what is the best treatment for adult bruxists?

**Methods/design:**

Comprehensive searches of the Cochrane Library, MEDLINE (via PubMed), Scopus, and LILACS will be completed using the following keywords: bruxism and therapies and related entry terms. Studies will be included, according to the eligibility criteria (Controlled Clinical Trials and Randomized Clinical Trials, considering specific outcome measures for bruxism). The reference lists of included studies will be hand searched. Relevant data will be extracted from included studies using a specially designed data extraction sheet. Risk of bias of the included studies will be assessed, and the overall strength of the evidence will be summarized (i.e., GRADE). A random effects model will be used for all pairwise meta-analyses (with a 95% confidence interval). A Bayesian network meta-analysis will explore the relative benefits between the various treatments. The review will be reported using the Preferred Reporting Items for Systematic Reviews incorporating Network Meta-Analyses (PRISMA-NMA) statement.

**Discussion:**

This systematic review aims at identifying and evaluating therapies to treat bruxism. This systematic review may lead to several recommendations, for both patients and researchers, as which is the best therapy for a specific patient case and how future studies need to be designed, considering what is available now and what is the reality of the patient.

**Systematic review registration:**

PROSPERO CRD42015023308

**Electronic supplementary material:**

The online version of this article (doi:10.1186/s13643-016-0397-z) contains supplementary material, which is available to authorized users.

## Background

Different definitions for bruxism have been proposed. The American Academy of Sleep Medicine, in 1990, defined sleep bruxism (SB) as a parasomnia because it is an undesirable physical phenomenon which occurs predominantly during sleep [1]. In 2010, another study defined sleep bruxism as the stereotyped oromandibular activity during sleep, characterized by teeth grinding and clenching [2]. In 2013, bruxism was also defined as the repetitive jaw-muscle activity characterized by clenching or grinding of the teeth and/or by bracing or thrusting of the mandible, in an international consensus [3]. The known manifestations of bruxism are sleep bruxism, which occurs during sleep, and awaking bruxism, which occurs during wakefulness [3]. Regardless of the definition, etiology or kind of manifestation, it is mainly characterized by teeth grinding and clenching, and patients diagnosed with this condition are commonly referred to as bruxists.

According to a recent review, both bruxism physiology and pathology have unknown causal associated factors. Nevertheless, some conditions like smoking, use of certain medications, and breathing problems can be considered as risk factors for bruxism [4]. Indeed, the main and widely accepted hypothesis is that the abnormal rhythmic mandibular movements detected during bruxism activity are caused by central and autonomic nervous system [5]. In the past, based on suspicion that occlusal imbalance was the main etiological factor for bruxism, dentists used to indicate occlusal adjustment [6], occlusal stabilization splints [7], or even oral rehabilitation, based on occlusal equilibration theories to deal with bruxists [8, 9]. These treatments, especially occlusal splints, still have no proven effectiveness for bruxism management based on RCTs and should be considered as a more limited treatment modality once the splints’ effect seem not to address the cause of bruxism and serves mainly for the management of patients’ signs and symptoms. [10, 11] Alternative therapies such as relaxation and biofeedback were proposed (and proved efficacious) for bruxism, especially in cases of awaking bruxism, which are more related to stress and anxiety [12–14].

Sleep hygiene techniques (e.g., relaxation before sleeping or avoiding caffeine) are also recommended to control sleep bruxism; however, recent data showed that these therapies were not efficacious for muscular activity control, once the autonomic muscular activity do not decrease using this sort of therapy [15]. The use of portable devices with contingent electrical stimulation (CES) is a promising strategy for bruxism therapy, especially because there are no side effects reported [16, 17]. The use of a night guard stabilization splint is also recommended after rehabilitation with dental implants, but no clinical trial confirmed this need [18–21]. There are also studies giving support to the use of clonidine and mandibular advancement appliances (MAA) and suggesting that occlusal splints should be used only as transient therapies while MMA can present side effects and maladaptation [7, 10, 18–21]. The MMA can actually be effective to reduce muscular activity in bruxists [22], but there is currently a lack of long-term evaluation. Both CES and MMA still need further investigation.

Botulinum toxin injection in the masticatory muscles is another promising treatment alternative to reduce muscular activity in bruxists, but scarce strong data exists to support this therapy as a routine, while side effects may also be present [23–25]. In fact, some drugs can be used to decrease bruxism episodes, but some pharmacological treatments may be unsafe if used for long periods, considering the inherent side effects or risks of dependency [7].

Interventions in individuals diagnosed as bruxists used to be needed to control pain, temporomandibular disorders, or in an attempt to control the progression of tooth wear [7, 19, 26]. These signs and symptoms can be caused or even enhanced by bruxism, but the current support therapies for bruxism aim mainly to control the consequences rather than to address to the cause(s) of the(se) problem(s) [27]. The multifactorial etiology of orofacial pain and temporomandibular disorders makes these problems not always solvable by the dentist alone, even when bruxism is suspected to be involved in the etiology.

### Review objectives

We plan to conduct a systematic review and network meta-analysis to try to answer the following question: which is the best treatment for adult bruxists, considering the reduction of muscular activity or reduction of grinding noises at night, sleep-related variables, comorbidities, and costs of the different therapies found. We will explore in this review as primary outcome parameters masseter muscle activity measurement using electromyography and slow wave sleep percentage during the polysomnographic recording.

### Why it is important to do this review

Bruxism is a common condition with a prevalence ranging from 8 to 31% in adults [28]. As it was shown, an effective long-term therapy to treat bruxists still lacks and several remaining questions still need to be answered, as “what should be done to support patients that are confirmed bruxists” or “what should be done after an oral rehabilitation is conducted and the patient is still clenching.” Some reviews, systematic reviews, and meta-analysis still show no definitive answers for all these questions [11, 12, 29], and this may be due to the comparison of only two interventions at a time, not summarizing a more comprehensive set of comparisons addressing the multiple interventions available. Remarkably, few studies compare different treatment effects of diverse current available therapies [7]. It would be helpful for the clinician and patients to know the current available treatments for the different manifestations of bruxism, what are their advantages and disadvantages to better choose among the options based on evidence and not only on expert’s opinion.

## Methods/design

The protocol of this systematic review and network meta-analysis will be written in accordance with the PRISMA-P (Preferred Reporting Items for Systematic Review and Meta-Analysis Protocols) [30] guidance. The completed systematic review will be written using the PRISMA-NMA extension statement to structure the contents of the final report. [31] This protocol is registered in the PROSPERO database (international prospective register of systematic reviews) as CRD42015023308. The literature search was established to address the research question phrased as follows in the PICO framework: Population—adult patients diagnosed with bruxism; interventions and comparisons: adenotonsilectomy, medications (benzodiazepines, dopaminergics, sympatholytics, antihistamines, antiepileptics, antidepressants, serotonin precursors), compared among them or to placebo; botulinum toxin intramuscular injections compared to saline solution, muscular electric stimulation (contingent, microcurrent or transcutaneous current), biofeedback (audible noise/sound, adverse taste response to muscular activity), behavioral (relaxation exercises, sleep hygiene measures, cognitive instructions), and kinesiotherapy (facial massage, masticatory or facial muscles’ exercises). It is possible in some reports that the therapies’ results might be compared among them, compared to placebo groups or to controls; outcomes—decrease of muscular activity (records with different kinds of electromyography), relief in muscular symptoms (e.g., pain, soreness, discomfort, fatigue, either self-reported or through clinical examination), decrease or arrest of dental wear, or dental grinding noises; study design—RCT and CCT. We will consider any published trial from 1956 to present published in the English language.

### Criteria for selecting studies for this review

The following criteria will be used to identify studies to be included in this review.

### Types of participants

Studies that enrolled adults (from 18 years of age) diagnosed as bruxists will be considered for inclusion. Studies including patients with tooth wear, temporomandibular disorders, or orofacial pain will also be eligible for inclusion.

### Interventions

According to the literature, interventions for bruxists are of wide variation and can be divided into the following groups: (1) intraoral: occlusal adjustment, occlusal splints, mandibular advancement appliances, NTI (nociceptive trigeminal inhibitory) splint; (2) physiotherapy for masticatory muscles’ with electrical stimulus: biofeedback, microcurrent transcutaneous stimulation, transcutaneous electrical nerve stimulation, contingent electrical stimulation; (3) drug therapy: antidepressants, l-dopa inhibitors, antiepileptic, sympatholytic, antihistamine, or dopaminergic drugs; (4) intramuscular injection: botulinum toxin A; (5) biofeedback: aversive taste, audible noise, or audible sound; (6) behavioral: relaxation techniques, “sleep hygiene” measures, cognitive treatment, psychological advice; (7) kinesiotherapy: masticatory muscles’ massage, facial exercise, or (8) others: alternative or support therapies. Any study evaluating any of the interventions listed above will be retained for inclusion in the review. Analysis will be conducted at therapy/group level.

### Types of studies

We will include randomized controlled trials (RCTs) and controlled clinical trials (CCTs). Review papers, expert opinions, case reports, and series of case reports will be excluded. The bibliographies of relevant systematic reviews will be studied to identify any studies missed by our literature search.

### Information sources and literature search

#### Electronic searches

Comprehensive searches of the Cochrane Library, MEDLINE (via PubMed), Scopus, and LILACS will be completed using the strategy described on the Additional file 1. Two independent authors (MEM and JAS) will select the articles. The search will encompass all the indexed articles, computerized literature databases supplemented by manual searching of reference lists from each relevant paper identified.

#### Study selection procedure

All titles and abstracts found will be independently read. After the searches, when found, the duplicates will be removed and the papers evaluated. The abstracts found in multiple searches to identify potentially eligible articles for inclusion will be read. All potentially eligible studies will be retrieved and full-text articles reviewed to determine eligibility. Hand search in the references of the selected studies will be also done. Inconsistencies will be solved by discussion among independent investigators (RSO and TC). In case of missing data or information, authors will be contacted. The reviewers that will be enrolled in the searches are experienced in orofacial pain management, specialist clinicians, or methodologists in evidence-based medicine.

#### Data collection process

A standardized, electronic data collection form implemented in Microsoft Excel will be used to extract the following data: study design, diagnosis, number of participants, types of interventions compared, patient demographics, outcome measures, results, risk of bias assessment, and study authors’ main conclusions. Two researchers will perform data extraction independently.

#### Outcomes

Different outcomes will be considered in this review whenever available:

- Primary effects: reduction of masticatory muscle activity (duration or intensity) detected by measurement of episodes per hour (day or nighttime) which can be assessed with polysomnography or in millivolts with electromyography;
- Secondary effects (measured, self-reported or reported by bed partners): sleep quality improvement, mouth range improvement, patient discomfort, temporomandibular joint pain (orofacial pain), sounds, psychological discomfort, quality of life, stress degree/level, tooth grinding reduction, dental wear arrestment;
- Comorbidity (side effects or adverse effects), costs, time span of the treatment (short-span and long-span outcomes can be evaluated separately) and;
- Compliance (adherence to the treatment) with the different therapies.

#### Heterogeneity assessment

The different manifestations of bruxism (awake or sleep bruxism) may be treated separately in the outcome analysis, considering the subgroup analyses and/or meta-regression.

#### Assessment of effectiveness

The tools used to verify the effectiveness of bruxism therapy are mostly electromyography (portable devices or non-portable devices) or electrodes used in polysomnography. According to the literature, a therapy is effective for bruxism when the episodes of masticatory muscle activity (masseter and temporalis) are reduced and, depending on the methods of the studies number of episodes per hour, numbers of episodes per night are used to quantify muscular activity. Duration and intensity of muscular activity can also be used to assess the effect of certain bruxism therapies.

Other parameters may be used to monitor reduction of bruxism such as reduction of grinding sounds, tooth wear arresting, and masticatory muscle pain.

#### Risk of bias assessment

Studies will be assessed for bias using the Cochrane risk of bias tool considering the judgment of the random sequence generation, allocation concealment, blinding of participants and personnel, blinding of outcome assessment, incomplete outcome data, selective reporting, and other sources of bias as “Low risk” of bias, “High risk” of bias, or “Unclear risk” of bias.

#### GRADE assessment

The evidence will be interpreted according to the GRADE Working Group approach for rating the quality of treatment effect estimates from network meta-analysis. This approach is based on four steps considering direct and indirect treatment estimates for each comparison of the evidence network, rating the quality of each direct and indirect effect estimate, rating the NMA estimate for each comparison of the evidence network and quality of each NMA effect estimate [32].

#### Data synthesis

An overview of all selected studies will be narratively displayed. Once data are obtained, a sheet will be made to tabulate data for the different outcomes. Classification according to the population and study characteristics and nature of the therapy will be made. Both traditional pairwise meta-analyses and network meta-analyses will be conducted.

#### Standard pairwise meta-analysis

A random effects model will be used for all pairwise analyses when data are available. The heterogeneity will be evaluated through the estimation of the variance between studies (chi-square test and *I* ^2^ statistic). We will split the muscle activity into reduction or non-reduction, and the number of people who were in non-reduction group will be used to calculate the odds ratios (OR) and corresponding 95% confidence intervals (CIs). Mean differences between treatments may also be considered.

#### Network geometry

The network of treatments will be judged based on the available study data presented and evaluated graphically. We will evaluate if there is a sufficient number of comparisons in network with no available data, if there is a high number of comparisons based on single studies, if there are any “closed loops” which allow testing agreement between direct and indirect estimates for comparison on network, if any key treatments are missing, and if the possible lumping of treatments is minimizing the clinical relevance of the review. Next, the feasibility of a network meta-analysis will be assessed. Treatments will probably be lumped into eight groups a priori (cited earlier) considering the type of treatment: intraoral, physiotherapy, drug therapy, intramuscular injection, biofeedback, kinesiotherapy, and other alternative or support therapies.

#### Network meta-analysis (NMA)

NMA will be performed using a Bayesian framework through the Winbugs software considering the random effects models, which use vague (noninformative) prior distributions for all treatment effects as well as the between-study variance parameter. The results of all pairwise comparisons will be reported as OR and corresponding 95% credibility intervals (CrIs). The median treatments rankings and the surface under the cumulative ranking curve (SUCRA) will be presented as well. Analyses will be performed using Markov-Chain Monte-Carlo methods.

We will assess the convergence based on the Gelman Rubin diagnostics and inspection of Monte Carlo errors [33]. The consistency of results will be assessed examining through the comparison of results of pairwise and network meta-analyses. Also, we will evaluate the consistency by fitting the consistency and inconsistency models for network meta-analyses and through the comparison of deviance information criterion (DIC) between both models with smaller values indicative of a better fit and considering a difference of 5 or more as important [34]. Possible violation of transitivity could be associated with inclusion of patients with different health conditions and different habits. We will explore this through a subgroup and meta-regression analyses considering the following factors: inclusion of patients with obstructive sleep apnea syndrome, loud snorers, subjects with moderate daytime sleepiness, heavy alcohol drinkers, caffeine drinkers, smokers, subjects with a highly stressful life, and those with anxiety.

## Discussion

This systematic review is planned as the current literature points toward the direction that the amount of wear is not always related to nocturnal bruxism activity [35]. If this is indeed correct, there is a void on what treating bruxism really means. Dentists and patients sometimes do not know if they are treating/being treated for muscular pain, tooth wear, or even if they are being protected against dental wear (anecdotal). Lastly, we feel that this systematic review may lead to several recommendations, for both patients and researchers, as which is the best therapy for a specific patient case and how future studies need to be designed [36], considering what is available now and what is the reality of the patient.

Although the comparison among treatments in a network meta-analysis is performed based on direct comparisons of interventions and indirect comparisons based on a common comparator, some factors may influence the estimates obtained from the analysis as number of trials included in the network; heterogeneity and inconsistency and several methods have been proposed to lead with those factors [37, 38]. In dentistry, this analytical approach has been used in different subjects such as restorative dentistry, periodontology, cariology and others [39–41].

## Additional file

Additional file 1: Search terms. (DOCX 35 kb)

## Abbreviations

CCT: Controlled clinical trials
CES: Contingent electrical stimulation
CI: Confidence intervals
CrIs: Credibility intervals
DIC: Deviance information criterion
GRADE: DIC
MAA: Mandibular advancement appliances
NMA: Network meta-analysis
NTI: Nociceptive trigeminal inhibitory
OR: Odds Ratios
PRISMA-NMA: Preferred Reporting Items for Systematic Reviews incorporating Network Meta-Analyses
PRISMA-P: Preferred Reporting Items for Systematic Review and Meta-Analysis Protocols
RCT: Randomized controlled trials
SB: Sleep bruxism
SUCRA: Surface under the cumulative ranking curve
