# Supplementary material for: Therapies for bruxism: a systematic review and network meta-analysis (protocol)
Source: Syst Rev. 2017 Jan 13;6:4. doi: 10.1186/s13643-016-0397-z (PMC5237268; doi:10.1186/s13643-016-0397-z)
Supplement: Additional file 1: — Search terms. (DOCX 35 kb) [file 13643_2016_397_MOESM1_ESM.docx]

**Pubmed**

**Total: 3205**

**Date: 11/07/2016**

**("Bruxism"[Mesh] OR "Bruxism" OR "Teeth Grinding Disorder" OR "Disorder, Teeth Grinding" OR "Disorders, Teeth Grinding" OR "Grinding Disorder, Teeth" OR "Grinding Disorders, Teeth" OR "Teeth Grinding Disorders")**
